# Wrist-Based Electrodermal Activity Monitoring for Stress Detection Using Federated Learning

**DOI:** 10.3390/s23083984

**Published:** 2023-04-14

**Authors:** Ahmad Almadhor, Gabriel Avelino Sampedro, Mideth Abisado, Sidra Abbas, Ye-Jin Kim, Muhammad Attique Khan, Jamel Baili, Jae-Hyuk Cha

**Affiliations:** 1 Department of Computer Engineering and Networks, College of Computer and Information Sciences, Jouf University, Sakaka 72388, Saudi Arabia; aaalmadhor@ju.edu.sa; 2 Faculty of Information and Communication Studies, University of the Philippines Open University, Los Baños 4031, Philippines; garsampedro@ieee.org; 3 Center for Computational Imaging and Visual Innovations, De La Salle University, 2401 Taft Ave., Malate, Manila 1004, Philippines; 4 College of Computing and Information Technologies, National University, Manila 1008, Philippines; mbabisado@national-u.edu.ph; 5 Department of Computer Science, COMSATS University, Islamabad 45550, Pakistan; 6 Department of Computer Science, Hanyang University, Seoul 04763, Republic of Korea; yejinkim@hanyang.ac.kr (Y.-J.K.); chajh@hanyang.ac.kr (J.-H.C.); 7 Department of Computer Science, HITEC University, Taxila 47080, Pakistan; attiqu.khan@hitecuni.edu.pk; 8 College of Computer Science, King Khalid University, Abha 61413, Saudi Arabia; 9 Higher Institute of Applied Science and Technology of Sousse (ISSATS), Cité Taffala (Ibn Khaldoun) 4003 Sousse, University of Sousse, Sousse 4000, Tunisia

**Keywords:** wearable sensor, privacy, federated learning, stress detection, healthcare, deep neural network

## Abstract

With the most recent developments in wearable technology, the possibility of continually monitoring stress using various physiological factors has attracted much attention. By reducing the detrimental effects of chronic stress, early diagnosis of stress can enhance healthcare. Machine Learning (ML) models are trained for healthcare systems to track health status using adequate user data. Insufficient data is accessible, however, due to privacy concerns, making it challenging to use Artificial Intelligence (AI) models in the medical industry. This research aims to preserve the privacy of patient data while classifying wearable-based electrodermal activities. We propose a Federated Learning (FL) based approach using a Deep Neural Network (DNN) model. For experimentation, we use the Wearable Stress and Affect Detection (WESAD) dataset, which includes five data states: transient, baseline, stress, amusement, and meditation. We transform this raw dataset into a suitable form for the proposed methodology using the Synthetic Minority Oversampling Technique (SMOTE) and min-max normalization pre-processing methods. In the FL-based technique, the DNN algorithm is trained on the dataset individually after receiving model updates from two clients. To decrease the over-fitting effect, every client analyses the results three times. Accuracies, Precision, Recall, F1-scores, and Area Under the Receiver Operating Curve (AUROC) values are evaluated for each client. The experimental result shows the effectiveness of the federated learning-based technique on a DNN, reaching 86.82% accuracy while also providing privacy to the patient’s data. Using the FL-based DNN model over a WESAD dataset improves the detection accuracy compared to the previous studies while also providing the privacy of patient data.

## 1. Introduction

Wearable technology has made significant strides in recent years. Humans can carry wearable devices such as smartwatches, spectacles, chest bands, and prosthetic implants [1]. In the larger area of the Internet of Medical Things (IoMT), wearable technology is simply one gadget. The IoMT comprises various medical tools and technologies that can collect and send data for medical use while connected. The IoMT also comprises ambient devices such as smart beds and detectors, implanted devices such as implantable cardiac and insulin pumps, and stationary devices such as hospital screens and imaging devices. Together, these gadgets gather and send information that can be used to track patient health, identify illnesses, and create specialized treatment regimens [2,3]. Accurate and persistent data analysis is possible with wearable technology, and this data can be utilized to track several elements of human health, such as stress levels. Wearable technology can help humans manage stress effectively by gathering data on irregular heartbeats, sleep habits, and physical activity [4].

Stress is a physical and physiological state that aggravates several serious illnesses, including diabetes, cardiovascular disease, and hypertension [5]. As reported by the British Health and Safety Executive, stress was the cause of 50% of all employment diseases in 2021–2022 (https://www.hse.gov.uk/statistics/causdis/, accessed on 23 January 2023). There exist two types of stress: Distress and Eustress. Distress is a harmful form of stress indicated by anxiety or a strong sensation of worry [6,7,8]. The consequences of distress are diminished performance and a fogginess of the mind. Further, eustress is a positive or beneficial form of stress that motivates us to achieve our goals, helps us to focus and concentrate, and keeps us alert and energized [9]. Furthermore, there are two categories of both Acute and Chronic. The main difference between acute and chronic eustress and distress is the duration and intensity of the stress and its impact on our health and well-being. Acute eustress is a short-term, positive stress that typically lasts for a short period. Acute distress is a short-term, negative stress typically lasting for a short period. Chronic eustress is a long-term, positive stress that typically lasts for a long period. Chronic distress is a long-term, negative stress that typically lasts for an extended period of time [10]. Severe or chronic illnesses might also lead to distress that is incredibly challenging for the body and brain to endure, ultimately resulting in depression and other problems with both physical and mental well-being [4,11,12]. Equally short-term and long-term occurrences are possible. Short-term stress might not always harm young and healthy individuals with an appropriate defense mechanism; however, if the traumatic situation is too frequent or severe, it may raise the chance of developing pathological conditions linked to anxiety and depression [13]. Acute periods of stress can precipitate a stroke or cardiac arrest, while long-term stress is recognized to raise the probability of life-threatening illnesses such as coronary artery disease, elevated cholesterol levels, diabetes, and adiposity [4].

In biomedical fields, self-reported questionnaires such as the Perceived Stress Scale (PSS) [14] are used to measure the psychological perception of stress and the State-Trait Anxiety Inventory (STAI) is used to measure responses to stress [15,16]. Further, there is evidence that stress-related anxiety can be detected through IMU sensors and video [17]. Following this strategy daily for regular inspection is not feasible because it requires time. Monitoring physiological responses to stress with sensors is another method for calculating stress levels [18,19,20]. The smartwatch is one of the best tools for stressful evaluations, particularly at work. Unlike certain wearables that are difficult to use and inconvenient to wear while working (such as chest-worn detectors, finger-placed galvanic skin response (GSR) sensors, etc.), the smartwatch is quite well recognized. It maintains a high level of social acceptability due to its widespread use in daily life [21]. The designed detectors of a smartwatch, such as an accelerometer, electrodermal activity, skin temperature, and blood volume pulse, can be employed for multimodal-based stress monitoring.

### 1.1. Motivation

The motivation for conducting this research is to automate wrist-based electrodermal activities and classification processes for timely and accurate stress diagnosis. ML technologies have enabled numerous useful applications of AI, including ones in the medical industry, with incredible success [22]. Previous research has extensively investigated the development of stress detection methods such as Decision Trees (DT), K-Nearest Neighbors (KNN), Random Forests, and Logistic Regression [23,24,25,26,27]. Machine learning approaches often need a large enough quantity of data to operate effectively during training. Hence, to develop a reliable approach, researchers must gather sensor information from many users and store it on a single server for analysis. However, the submitted medical data may present private and sensitive information about specific individuals. In cases where the cloud service is compromised, invasions of privacy may occur [28,29,30,31,32]. Information leakage can also occur when well-meaning individuals with privileged server access exchange information for legal causes [33,34]. As a result, increasing research emphasizes protecting personal information during the analytic process [35,36]. Federated learning (FL) potentially provides a solution for this privacy breach [37,38,39]. For FL to function, each data record must be free to train models on independent, separate datasets and then share only the completed, anonymized models. The models are then sent via the recorders to a centralized server, amalgamating into one integrative system. This process is repetitive until an elevated model is generated [40,41,42,43,44,45].

### 1.2. Contribution

This research contributes to more accurate and effective stress detection. The main contribution and features of the research are given below in the listed form.

- The study proposes a federated learning framework based on a deep neural network for predicting stress, incorporating data from wrist-worn sensors that train every client’s local model before sending the model parameters to the global model. The global model then aggregates the new parameter and initiates the training process, ensuring the privacy of the client’s data;
- The study collects the WESAD dataset from the UCI ML repository, which contains five states: transient, baseline, stress, amusement, and meditation. The raw data is converted into an acceptable format for the proposed framework using the preprocessing techniques SMOTE and normalization;
- The experiment reveals that the FL-based DNN model enhances stress detection accuracy compared to traditional approaches and provides privacy to the clients’ data.

### 1.3. Organization

This study work is organized into the following sections. The most current and relevant work on wearable sensor-based techniques, machine and deep learning, and federated learning in the healthcare field is given in Section 2. The proposed framework, which covers dataset selection, preprocessing, and model architecture, is described in Section 3. The evaluation measurements, findings, and results of the proposed approaches are described in Section 4. Finally, Section 5 includes the conclusion of the proposed approach and provides recommendations for more research.

## 2. Literature Review

The background and current research of federated learning, machine and deep learning, and wearable sensor-based techniques in the healthcare field are presented in this section.

### 2.1. Wearable Sensor-Based Techniques

With the most significant innovations in wearable technology, there is a substantial interest in continually monitoring stress using various physiological factors [46,47]. Ref. [48] addresses the connections between suffering and stress, how to measure them, and ways to identify them using wearable sensors and diagnostic implants. Numerous physiological signals, comprising pulse rate, neural activity, muscular activity, electrodermal action, breathing rate, blood volume pulse, and skin conductance, are structured to detect wearable sensors. The authors intend to discover a method for wearable health services systems to be employed in stress and pain assessment by analyzing the wearable sensors utilized in the healthcare system.

Ref. [26] examined which detector third-party producers have accessibility to and which ones are incorporated into the wearable technology that are now available in the marketplace. It investigates how the different study participants’ diagnostic accuracy differs from one another and what impact bandwidth has on the detection rate. The publicly accessible WESAD dataset is the foundation for each experiment. The findings demonstrate that an electrodermal movement sensor signal is not required for consumer stress detection and that, provided the interval length is large enough, consumer smartwatches may be utilized to diagnose stress. It should also be emphasized that the study participants’ detection rates vary considerably. The suggested system [49] classifies stress levels using multimodal data from the wearable Empatica E4 device on the wrist. The authors utilized four classification situations: stress, baseline, meditation, and amusement. The research used three classification techniques: Random Forest (RF), Logistic Regression (LR), and Decision Tree (DT). By measuring the effectiveness of the system, the researchers demonstrate that our approach can reliably identify the stress levels of 15 subjects with an accuracy of 88–99% using the RF.

Ref. [1] discussed ML objectives that have been studied in the context of wearable healthcare technology, the ML methods employed, the various modalities employed, and the relevant datasets. ML applications on wearable technology face various difficulties, including deployment options, energy usage, storage and memory requirements, functionality and user satisfaction, data availability and dependability, interaction, protection, and privacy concerns. These issues were discussed, along with the potential solutions that have been reported.

### 2.2. Machine and Deep Learning Techniques

The research provides various ML and DL algorithms for stress detection on persons utilizing heterogeneous datasets gathered through wearable metabolic and motion sensors to prevent a human from experiencing numerous stress-related health issues [50,51,52]. The WESAD dataset is employed in the ML and DL techniques. The accuracy for three-class (stress, amusement, and baseline) and binary (stress or non-stress) categories were compared using ML techniques. In addition, a feed-forward DL algorithm is introduced for these three-class and binary categories. The accuracy was reached for three-class and binary categorization issues using ML approaches up to 81.65% and 93.20%, respectively, while accuracy was obtained for these problems up to 84.32% and 95.21%, using DL [50].

In Ref. [53], the researchers strive to further emotion and sentiment evaluation to determine an individual’s level of stress based on the remarks and posts that a person has published on social media platforms. The authors use ML algorithms and a DL model called BERT for classification tasks to do sentiment analysis on massive data of tweets. The results show that the trained model can identify the emotional status based on social connections, which are assessed using multiple indicators at the level of the micro and macro. Models have been refined to classify emotions into joy, sadness, neutrality, anger, and fear. The accuracy of the categorization ability in NLP backed by deep contextual language models such as BERT was 94%, according to the researchers. In Ref. [4], the authors provided a thorough review emphasizing stress recognition utilizing wearable sensors and applicable machine learning methods. This study examines the methods used to identify stress with sensing devices, including wearable sensors, Photoplethysmography (PPG), Electrocardiograms (ECG), and Electroencephalograms (EEG), as well as in different contexts, such as while driving, learning, and working.

For stress detection, Ref. [54] created two DNNs: a Multilayer Perceptron (MLP) neural network and a 1-dimensional (1D) Convolutional Neural Network (CNN). The DNN examined physiological data obtained from the wrist and chest-worn devices to complete two activities. In the first activity, networks distinguished among stressed and non-stressed states using binary classification for stress detection. The networks employed a three-class classification task in the second experiment to distinguish between stressed, baseline, and amused states. For binary and 3-class classification, the deep MLP-NN attained an average accuracy of 99.65% and 98.38%, correspondingly. For binary and 3-class classification, the deep CNN attained accuracy rates of 99.80% and 99.55%, respectively. Ref. [55] proposed the convolutional neural network multi-level deep neural network with hierarchical learning capabilities. The WESAD benchmark dataset for mental health is used to evaluate the model, which compares favorably to cutting-edge methods and has an exceptional performance accuracy of 87.7%. Ref. [24] used different ML algorithms. The Random Forest model fared better than other models for the classification of binary and three classes, with F1 scores of 83.34 and 65.73, respectively.

### 2.3. Federated Learning Techniques

In Ref. [35], the authors focused on implementing federated learning-based stress identification and comparing individual, centralized, and federated learning. The WESAD dataset was used for the experiment, and the classifier used was Logistic Regression (LR). The personalized learning technique performed better, with an average F1-measure and accuracy of 0.9998 and 0.9996, respectively. The  findings of the experiment demonstrate that federated learning needs more individual and centralized accuracy. The researchers used federated learning in IoT-based wearable biomedical tracking devices to protect data privacy with positive outcomes. To track stress levels during various situations, Ref. [56] suggested federated learning to cardiac data information obtained using smart bands. Although the accuracies from these local models are 81.65% and 84.08%, respectively, we were able to reach 87.55% accuracy by integrating Datasets 1 and 2. Based on these findings, the contribution of the case study would be valuable for research organizations and businesses in the IoT-based wearable biomedical tracking field and may be used to address data security and privacy issues in data processing.

With the emergence of IoT technology, wearable devices are becoming popular for health monitoring and activities such as heart rate monitoring, medicine timing, pulse rate monitoring, sleep, walking, etc. [28,37,57,58]. ML models keep improving the performance of these devices without protecting the data. To address this issue, a framework FedHealth is proposed in Ref. [41] for wearable healthcare devices using federated learning. FedHealth uses federated learning for data aggregation and transfer learning for building models. FedHealth accuracy increased by 5.3% compared to other models while keeping data privacy.

In summary, different ML and DL techniques have been studied that provide techniques for predicting stress. However, they failed to provide encouraging results about greater accuracy and did not focus on securing data privacy. FL studies focused on overcoming the different healthcare issues and system privacy [40,41,42,43,44,45]. Hence, in this study, an FL-based DNN model is proposed to ensure data privacy that ML and DL models are not considering.

## 3. Proposed Model

**Figure 1:**
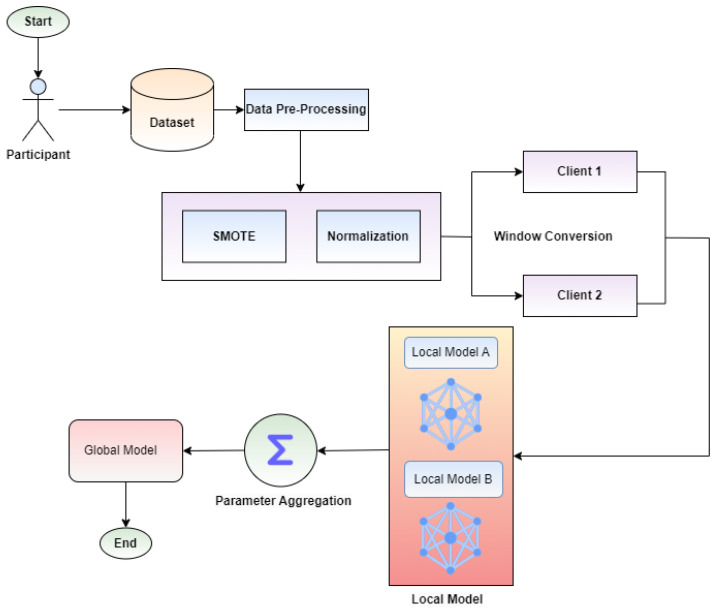
Proposed federated learning framework overview.

Privacy preserved automated wrist-based electrodermal activities classification for stress detection is the need of the time. To preserve privacy and detect stress, this section represents the fundamental concept of the proposed methodology, including federated learning, model design, and data preprocessing. Figure 1 depicts the training process of the local and global model with the WESAD (Wearable Stress and Affect Detection) dataset using a DNN model, which consists of the following steps. First, data preprocessing of the selected dataset is performed using SMOTE and normalization. Second, the preprocessed dataset is split into two windows for two clients. Then the local model is trained using a DNN on the preprocessed dataset. The results of both clients aggregate and update the global model.

### 3.1. Federated Learning Framework

Three essential steps are included in the Federated Learning-based paradigm. Training Initialization was carried out in the first phase. The cloud-based federated learning (FL) server configures the required data type and trains the model parameters, including the number of epochs to be used, their value, Learning Rate (LR), and Activation Function (AF) [59,60]. The (FL) server also creates the initial global model. The DNN model that considers clients here receives specifications and model parameters. In addition to numerous hyper-parameters, the DNN model has specified client specifications. The federated learning server determines the epoch and learning rate of the model.

The DNN model needs to be trained at the second level. Every client begins by gathering new information and updating the local model’s ${(M}^{y}x)$ parameter, which is reliant on the global model ${(G}^{y}x)$, where y is the index for the subsequent iteration. Every client searches for the ideal scenario to reduce the loss. Finally, we provide the federated learning server with the new parameters regularly. Integration of the global model is the third stage. Once the results from several clients have been combined on the server side, we provide the revised parameters to each client. The federated learning server reduces the global mean loss function using Equation (1).

(1) $$Loss(G^{y})=\frac{1}{M}\sum_{x=M}^{x=1}Loss(M^{y}x)$$

In Algorithm 1, the dataset is first preprocessed $D_{s}$ and we increase the minority samples using SMOTE, apply min-max normalization, and partition the training dataset for two clients. In the second step, we update the global model and initialize the model weight $w_{0}$. ti denotes the current round of the model, and *T* is the total round of the local model. ci is the current client, and *C* is the total clients. We update the local model for each client according to the current iteration/round in the third step. The current iteration weight is calculated in the next step by the sum of the weight of the dataset of the client and the current client iteration. The following model parameters, such as an epoch value, activation function, and batch size, are used to calculate the loss of the local model of each client. We update the local model by calculating the loss function $F_{i}\left(w\right)$. The procedure is repeated until the requisite accuracy is attained or the loss function is constantly minimized.
**Algorithm 1** Federated learning framework for stress detection  1:$D_{s}$ (WESAD dataset)  2:$D_{p}$ (SMOTE and Normalization)  3:**function** global model upgrade  4: **weight initialization $w_{0}$**  5: **for** (ti=1) **to** T **do**  6:  **for** (ci=1) **to** C **do**  7:   $w_{ti+1}^{ci}$ = local model upgrade(ci,$w_{ti}$)  8:  $w_{ti+1}=\sum_{ci=1}^{C}$$w_{D_{s}}^{ci}$ * $w_{ti+1}^{ci}$  9:**function** local model upgrade (ci, $w_{ti}$)10: **for** (Epoch=1) **to**$E_{p}$ **do**11:  **for** ($B_{s}\in BatchSize$) **do**12:  $w=w-▽F_{i}\left(w\right)$13: **return** *w*

### 3.2. Experimental Dataset

This study used the Wearable Stress and Affect Detection (WESAD) [23], a publicly accessible UCI machine learning repository dataset. This study used the wrist-worn Empatica E4 to collect data from 15 individuals (https://www.empatica.com/e4-wristband, accessed on 23 January 2023). This gadget has accelerometers (ACC) and sensors for blood volume pulse, skin temperature, electrodermal activity, heart rate, and heart rate variability (HRV). WESAD additionally uses data from the chest-worn RespiBAN sensor in addition to E4 data, and participants also completed questionnaires about their emotions during the data collection session. The study emphasizes wrist-worn devices; thus, this research primarily uses E4 data. Data from five different affective states (transient = 0, baseline = 1, stress = 2, amusement = 3, and meditation = 4) were acquired during the data collection. The dataset has 8 attributes and contains 120,000 samples, of which 75% of data is used for training models, while the remaining 25% is used to assess the performance of the model.

### 3.3. Data Preprocessing

Data Preprocessing is a crucial step that improves the data quality to facilitate the extraction of analytical information from the data [61]. Real-world data frequently needs precise attribute values or trends that are more consistent, accurate (contains errors or outliers), and complete. In machine learning, data preparation converts raw data into a format appropriate for creating and refining machine learning models. Data preparation is the initial step in the machine learning process when building a model. Since it makes it easier to organize, clean, and prepare raw data so that machine learning models can use it, data preparation is crucial.

Two steps are used in data preprocessing to make raw data in an acceptable format for machine learning and deep learning models: SMOTE and Normalization. The min-max normalization method is applied to the preprocessing of data. Using low variance, the ambiguous dataset is structured, and data integrity is maintained using min-max scaling for normalizing the features. The input attribute scaling is crucial for a particular model that depends on the magnitude of values. As a result, normalizing describes the discrete range of real-valued numerical properties between 0 and 1. Equation (2) is being used for data normalization.

(2) $$Z_{norm}=\frac{Z_{i}-Z_{min}}{Z_{max}-Z_{min}}$$

SMOTE is an oversampling technique that represents the minority classes by using fictional samples. This method aids in overcoming the overfitting issue brought on by random oversampling. The total training data shape for client 1 before applying SMOTE techniques is 196,971, and when SMOTE is applied, the total training data shape is 143,781. The total training data shape for client 2 before applying SMOTE techniques is 200,000, and when SMOTE is applied, the training data shape remains the same at 200,000.

Following preprocessing, the dataset is split into two sections: a training dataset and a testing dataset, with 25% of the dataset used as testing data to assess the proposed model and 75% of the dataset used to train the model.

### 3.4. Model Architecture

This research examined the potential of DNN to detect stress by monitoring wrist-based electrodermal activities. The domain of learning that uses nonlinear information in different steps through hierarchical structures is included in the deep learning technique, as previously mentioned by Dong and Deng [62]. Deep learning is typically employed primarily for learning and pattern identification. Higher-level principles in the hierarchy of deep architecture are built using the lower-level components. Deep learning combines pattern recognition, neural networks, and visual design. For prediction on large datasets, the deep learning model works well. The proposed deep learning model analyzes the natural patterns in the diagnosis of stress.

**Figure 2:**
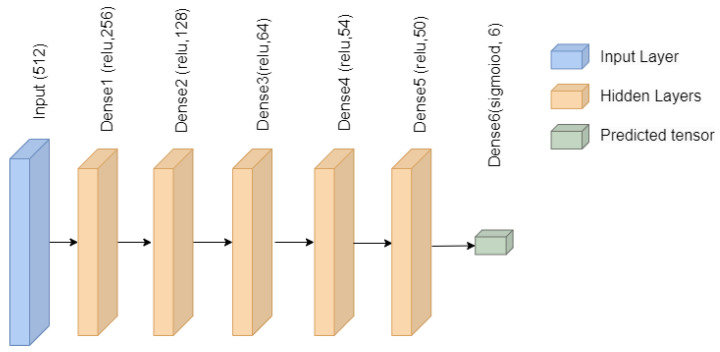
Deep neural network architecture.

This research uses a sequential DNN model with one input layer. The shape of the input layer is 8 with 512 units and uses the relu activation function. The hidden layer is the next, comprising five dense layers. The four dense layers comprise 256, 128, 64, 54, and 50 units, and the activation function is the relu. The output layer is next with 6 units and uses the softmax function. To address the categorical classification issue, every dense layer uses the activation functions relu and softmax along with a fully connected layer. The DNN model has used Adam as an optimizer to calculate and reduce the loss using sparse_categorical_crossentropy. The architecture of the DNN model is depicted in Figure 2.

## 4. Experimental Results and Analysis

The experiment is conducted on a WESAD dataset, which is available at the UCI machine learning repository. The dataset is split into two sets; each client uses a 50% dataset. Three rounds and five epochs for each round are set for both clients. The experimental results and an assessment of the suggested approach are provided in this section. The study also explores the impacts of several methodology-proposed parameters.

### 4.1. Evaluation Metrics

The prediction and classification issues are assessed using a variety of measures, such as accuracy, F1-score Recall, and precision. The following evaluation measures how well the proposed model works. To assess the precision of the suggested model, we compute the proportion of False Positives (FP), True Positives (TP), True Negatives (TN), and False Negatives (FN). Equation (3) represents the accuracy estimate. The proportion of actual positives to all positives in the data (false and true) is alternatively known as a highly anticipated value. Equation (4) presents the precision rate. The ratio of real positives to true positives and false negatives in a dataset is described using sensitivity, the likelihood of detection, and the probability of a genuine positive. Equation (5) indicates the recall rate. The weighted average of Recall and precision is the F1-score. The F1-score is provided in Equation (6).

(3) $$Accuracy=\frac{TP+TN}{TP+TN+FP+FN}$$

(4) $$Precision=\frac{TP}{TP+FP}$$

(5) $$Recall=\frac{TP}{TN+FN}$$

(6) $$F1\text{-score}=2\times \frac{Precision+Recall}{Precision+Recall}$$

### 4.2. Server-Based Training Using Log Data

The federated learning framework includes two clients and one server as its main parameter. In an introductory level of the model training phase, the server selects which client or node is used and aggregates any updates acquired. The DNN model is trained using server log data. The anonymized and individually identifying information is eliminated from the logs before training. There are three rounds after the federated learning begins. Before setting the number of rounds to 3, which includes evaluating the tests three times, researchers configure a few server-side parameters. The clients send the learning outcomes to the server during the fit-round. When the findings are combined in the assessment phase, both clients send the test results to the server. According to the server’s interpretation of the information gathered from N clients, the DNN model has the highest accuracy, scoring 86.21%.

### 4.3. Client 1 Federated Training and Testing

Table 1 summarizes the performance metrics of a suggested model for Client 1 in Round 1. The suggested model was evaluated in five classes: Transient, Baseline, Stress, Amusement, and Meditation. The Precision (P), Recall (R), and F1-score (F1) were computed for each class, along with the Support (S), which shows the number of samples in each class. The table also provided the model’s overall accuracy, macro Average, and weighted average. The model yielded high performance in all classes, with F1-scores ranging from 98% to 100%. The weighted average F1-score was 99%, indicating that the model performed well across all classes, considering the differences in class distribution. The model obtained the highest F1-score on the Baseline class, with outclassed Precision and Recall. It suggests that the model is particularly good at identifying this class. Overall, the model achieved an Accuracy of 99%, which means that it correctly classified 99% of the samples. The Macro Average and Weighted Average F1-scores were also high, at 98% and 99%, respectively. It indicates that the model outperforms classifying all classes and is particularly fine at detecting the dominant classes.

Table 2 represents the performance metrics of a proposed model for Client 1 in Round 2. As in Round 1, the model was evaluated in five classes: Transient, Baseline, Stress, Amusement, and Meditation. The Precision (P), Recall (R), and F1-score (F1) were computed for each class, along with the Support (S), which represents the number of samples in each class. The table also shows the model’s overall Accuracy, Macro Average, and Weighted Average. The proposed model outperformed in all classes, with F1-scores ranging from 97% to 100%. The weighted average F1-score was 99%, indicating that the model outclassed all classes, considering the differences in class distribution. The model achieved the highest F1-score on the Baseline class, with exceptional Precision and Recall, similar to the results in Round 1. Compared to Round 1, the model achieved a slightly lower F1-score on the Stress class, which decreased from 98% to 97%. However, this is still a high F1-score, and the Precision and Recall for this class are also high. The model achieved the highest Recall in the Transient class, with a score of 99%, which means it correctly diagnosed most of the samples in this class. Overall, the model achieved an Accuracy of 0.99, meaning it accurately classified 99% of the samples. The Macro Average and Weighted Average F1-scores were also high, at 99%, indicating that the model is exceptional at categorizing all classes and is particularly good at identifying the dominant classes.

Table 3 summarizes the results of round 3 for client 1 by monitoring the wrist-based electrodermal activities to detect stress. The table contains information about the precision (P), Recall (R), F1-score (F1), and support (S) for each class and the overall evaluation metrics such as accuracy, macro average, and weighted average. The support represents the number of instances for each class, while the accuracy represents the correct overall predictions made by the model. The macro average and weighted average are used to evaluate the model’s performance across all classes, where the macro average gives equal weight to each class, and the weighted average considers the number of instances for each class. The results show that the model’s performance improves in round 2 compared to round 1, but there is a slight drop in performance for the Amusement class in round 3. Overall, the model has high accuracy and performs well for all classes, with F1-scores ranging from 98% to 99% for each class.

**Figure 3:**
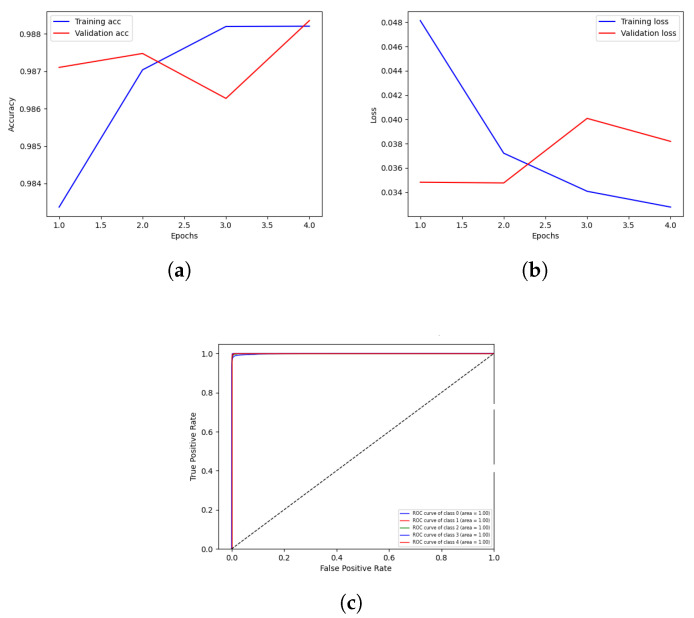
Graphical visualization of client 1 highest-scoring outcome. (**a**) Validation and training accuracy of round 3; (**b**) Validation and training loss of round 3; (**c**) Receiver Operation Characteristic score of round 3.

The highest results of client 1 are illustrated in Figure 3. The graph in Figure 3a shows the training and validation accuracy, with round 3 of client 1 achieving higher validation accuracy than training accuracy. A blue line shows the training accuracy curve, while an orange line shows the validation accuracy curve. At the 1th epoch, the training accuracy is 0.983%; after various fluctuations between falls and gains, it reached about 0.988% accuracy at the 4th epoch. At the 1th epoch, validation accuracy is 0.987%; it then fluctuates between drops and gains reached at 0.99% accuracy at the 5th epoch.

The training and validation loss is depicted on the graph in Figure 3b. During the training phase, the loss fluctuates at each epoch. The blue line shows the training loss curve, while the orange line represents the validation loss in the loss curve. Training loss initiated from 0.048% at 1th epoch and decreased to 0.033 at 4th epoch. Validation loss initiated from 0.034% at 1th epoch and increased to 0.038% at 5th epoch. The Receiver Operating Characteristic (ROC) is depicted in the graph in Figure 3c. AUCROC score is 1.00%, indicating that DNN-based FL has effectively classified the data into the five categories of transient, baseline, stress, amusement, and meditation. The ROC curves close to the top-left corner demonstrate better performance.

### 4.4. Client 2 Federated Training and Testing

Table 4 shows the performance metrics of a proposed model for classifying data from Client 2 in Round 1. The classes are Transient, Baseline, Stress, Amusement, and Meditation. The performance of the model is measured using Precision (P), Recall (R), and F1-score (F1) for each class, as well as the total number of samples in each class (Support or S). The table also includes the overall Accuracy, Macro Average, and Weighted Average of all classes. According to the results, the model achieved high precision, Recall, and F1-score for most classes, indicating good performance in classifying the data. The model yielded an overall 98% accuracy. The Transient class has the highest F1-score of 98%, with 35,183 samples. The Baseline, Stress, Amusement, and Meditation classes also achieved high F1-scores ranging from 97% to 98%. The model achieved an overall F1-score of 98%, indicating good performance in classifying all the classes. The Macro Average and Weighted Average also achieved high F1-scores of 97% and 98%, respectively, indicating the model’s overall performance.

Table 5 shows the result of the proposed model for Client 2 in Round 2. The performance of the model is evaluated using Precision (P), Recall (R), F1-score (F1), and Support (S) metrics for each class. The table also includes Macro Average and Weighted Average scores. The suggested model achieved a high F1-score of 98%, which indicates good overall performance. Among the five classes, the Transient class has the highest precision and Recall scores of 99% and 98%, respectively. The Baseline, Stress, Amusement, and Meditation classes also have high precision, Recall, and F1 scores, ranging from 95% to 99%. The average macro score for precision, Recall, and F1 is 97%, 98%, and 98%, respectively, which indicates a good overall performance of the model across all classes. The weighted average score is also high, which suggests that the model is performing well overall, considering the class imbalance in the dataset.

Table 6 presents the performance metrics of a proposed model for Client 2 in Round 3. The model was evaluated on five classes (Transient, Baseline, Stress, Amusement, and Meditation) using precision (P), recall (R), F1-score (F1), and support (S) metrics. The accuracy, macro average, and weighted average were also calculated. The results represent that the proposed model achieved high precision, Recall, and F1-score for all five classes, with F1-score ranging from 98% to 99%. The highest F1-score was obtained for the Transient class, which had precision and recall values close to 100%. The model also achieved high accuracy of 98% and performed well on both macro average and weighted average metrics.

**Figure 4:**
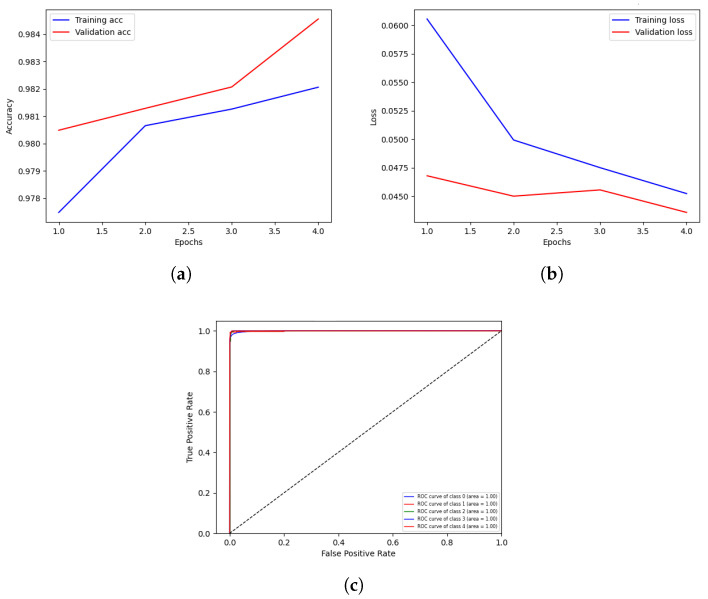
Visualization of client 2 highest-scoring results. (**a**) validation and training accuracy of round 3; (**b**) Validation and training loss of round 3; (**c**) Round 3 score of Receiver Operation Characteristic.

The highest results are illustrated in Figure 4. The graph in Figure 4a shows the training and validation accuracy, with round 3 of client 2 attaining higher validation accuracy than training accuracy. A blue line shows the training accuracy curve, while an orange line represents the validation accuracy curve. At the 1th epoch, the training accuracy is 0.977%; after various fluctuations between falls and gains, it reached about 0.981% accuracy at the 4th epoch. At the 1th epoch, the validation accuracy is 0.980%; however, it fluctuates between drops and gains until reaching 0.984% accuracy at the 4th epoch. The training and validation loss is depicted on the graph in Figure 4b. During the training phase, the loss fluctuates at each epoch. The blue line represents the training loss curve, while the orange line represents the validation loss in the loss curve. The training loss initiated from 0.060% at 1th epoch and decreased to 0.045 at 4th epoch. The validation loss initiated from 0.047% at 1th epoch and decreased to 0.042% at 5th epoch.

The Receiver Operating Characteristic (ROC) is depicted in the graph in Figure 4c. The experiment uses five classes, and the proposed model performs better on the used dataset with ROC scores of 1.00%. The ROC curves near the top-left corner demonstrate better performance, indicating that DNN-based FL has effectively classified the data into the five categories of transient, baseline, stress, amusement, and meditation.

**Figure 5:**
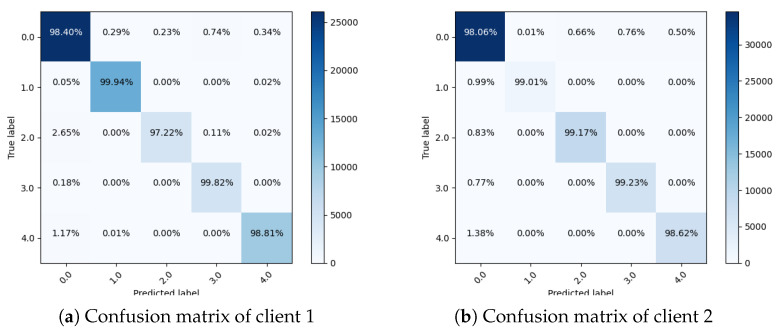
Confusion matrices for both clients using proposed method.

Figure 5 graphically visualize the confusion matrix of both clients. The confusion matrix of Client 1, displayed in Figure 5a, indicates that 98.40% transient cases are predicted successfully, whereas 1.6% cases are misdiagnosed. Similarly, out of 13,191 baseline cases, 99.94% are predicted accurately, 0.07% are misdiagnosed, and out of 4713 stress instances, 97.22% are predicted successfully, while 2.78% instances are misclassified. Similarly, out of 4891 amusement cases, 99.82% are predicted correctly, 0.18% are misdiagnosed, and in 9789 meditation instances, 98.81% are predicted successfully, while 1.17% instances are misdiagnosed. The confusion matrix of Client 2, shown in Figure 5a, indicates that 98.06% transient cases are predicted successfully. Similarly, 99.01% baseline cases are predicted accurately, 0.99% are misdiagnosed, and out of 9046 stress cases, 99.17% are predicted successfully, while 0.83% cases are misclassified. Similarly, 99.23% of amusement cases are predicted correctly, whereas 0.77% are misdiagnosed, and 98.62% of meditation instances are predicted successfully, whereas 1.38% instances are misdiagnosed.

## 5. Discussion and Comparative Analysis

This research focused on preserving the privacy of patient data while classifying wearable-based electrodermal activities. The Federated Learning (FL) based Deep Neural Network (DNN) model is used to preserve privacy and improve the performance of stress detection. The comparison of the proposed model with previous techniques is provided in Table 7. The authors in Ref. [55] used a CNN model, achieving an accuracy of 87.7/%. The researchers in Ref. [24] utilize ML techniques and achieved an F1-score of 83.34. The authors in Ref. [23] also use ML techniques, achieving an accuracy of 80.0%. The proposed approach uses a federated learning-deep neural network (FL-DNN) that achieves an accuracy of 99.1%, precision of 98.0%, recall of 97.0%, and F1-score of 97.0%. The results revealed that the proposed approach helps identify stress in people earlier, assisting medical practitioners in more timely and accurate stress detection.

### Research Limitations

The sustainability of healthcare systems relies on using user data to train ML models while raising severe privacy and security concerns. To address this issue, this research proposes an FL-based DNN model to recognize and classify wearable electrodermal activities. Since FL prioritizes protecting patient data, no direct data exchange occurs in this collaborative process. Although FL permits more collaborative ML (with privacy protection at its core), it also presents many problems, including those mentioned here. Communication is a significant hurdle in FL networks, as the data created by each device is kept locally. Designing models that allow effective communication while limiting the number of cycles to train a model using device-generated input is crucial. Additionally, it should incrementally communicate discrete model upgrades rather than providing the complete dataset during training. Although FL’s privacy features are a big positive, they also prevent data analysts from seeing user data in its unprocessed form. To detect missing data, eliminate extraneous information, and identify the data points the system should be trained on, they cannot clean up the data, resulting in the system’s poor performance. Furthermore, another limitation of the stress detection dataset is that even though the WESAD data induced distress, using data such as these to detect stress does not allow us to identify eustress and distress.

## 6. Conclusions

This study proposed the wrist-based electrodermal activities and federated learning framework on the WESAD dataset. Despite a slight variance in accuracy, the proposed model correctly identified stress from the provided data. While protecting client data, the suggested FL-based DNN performed exceptionally well in identifying the classes. To reduce the effect of over-fitting, the outcomes for each client are validated three times. The DNN model is successively trained on the dataset after a potential boost by two clients and aggregated at the server side with an accuracy of 86.82%. The study concludes that Wearable technology makes it possible to analyze data accurately and persistently, and this data can be used to measure various aspects of human health, such as stress levels. By monitoring irregular heartbeats, sleep patterns, and physical activity, wearable devices can assist people in efficiently managing stress. The earlier a disease is diagnosed, the simpler it is to treat it. The proposed research can identify these alterations in people earlier, assisting medical practitioners in a more timely and accurate stress detection. We plan to investigate this trend more thoroughly by training more systems using various wearable device combinations and expanding our research by applying new deep learning algorithms with multiple datasets and employing different statistical tests, such as Wilcoxon and ANOVA, in the future to ensure the quality of the proposed algorithm.

## Figures and Tables

**Table 1:** Proposed model result of Client 1 for round 1. Key: Precision-P; Recall-R; F1-score-F1; S-Support.

Classes	P	R	F1	S
Transient	0.98	0.99	0.98	26,508
Baseline	0.98	1.00	1.00	13,191
Stress	0.98	0.97	0.98	4713
Amusement	0.99	0.98	0.98	4891
Meditation	0.99	0.97	0.98	9789
Accuracy	-	-	0.99	59,092
Macro Avg	0.99	0.98	0.98	59,092
Weighted Avg	0.99	0.99	0.99	59,092

**Table 2:** Proposed model result of Client 1 for round 2. Key: Precision-P; Recall-R; F1-score-F1; S-Support.

Classes	P	R	F1	S
Transient	0.99	0.98	0.98	26,508
Baseline	0.99	1.00	1.00	13,191
Stress	0.99	0.96	0.97	4713
Amusement	0.98	0.98	0.98	4891
Meditation	0.99	0.99	0.99	9789
Accuracy	-	-	0.99	59,092
Macro Avg	0.99	0.98	0.99	59,092
Weighted Avg	0.99	0.99	0.99	59,092

**Table 3:** Proposed model result of Client 1 for round 3. Key: Precision-P; Recall-R; F1-score-F1; S-Support.

Classes	P	R	F1	S
Transient	0.99	0.98	0.99	26,508
Baseline	0.99	1.00	1.00	13,191
Stress	0.99	0.97	0.98	4713
Amusement	0.96	1.00	0.98	4891
Meditation	0.99	0.99	0.99	9789
Accuracy	-	-	0.99	59,092
Macro Avg	0.98	0.99	0.99	59,092
Weighted Avg	0.99	0.99	0.99	59,092

**Table 4:** Proposed model result of Client 2 for round 1. Key: Precision-P; Recall-R; F1-score-F1; S-Support.

Classes	P	R	F1	S
Transient	0.99	0.97	0.98	35,183
Baseline	0.95	0.99	0.97	1515
Stress	0.98	0.97	0.97	9046
Amusement	0.95	0.99	0.97	6746
Meditation	0.96	0.98	0.97	7510
Accuracy	-	-	0.98	60,000
Macro Avg	0.97	0.98	0.97	60,000
Weighted Avg	0.98	0.98	0.98	60,000

**Table 5:** Proposed model result of Client 2 for round 2. Key: Precision-P; Recall-R; F1-score-F1; S-Support.

Classes	P	R	F1	S
Transient	0.99	0.98	0.98	35,183
Baseline	0.97	0.99	0.98	1515
Stress	0.97	0.98	0.98	9046
Amusement	0.95	0.99	0.97	6746
Meditation	0.99	0.97	0.98	7510
Accuracy	-	-	0.98	60,000
Macro Avg	0.97	0.98	0.98	60,000
Weighted Avg	0.98	0.98	0.98	60,000

**Table 6:** Proposed model result of Client 2 for round 3. Key: Precision-P; Recall-R; F1-score-F1; S-Support.

Classes	P	R	F1	S
Transient	0.99	0.98	0.99	35,183
Baseline	1.00	0.99	0.99	1515
Stress	0.97	0.99	0.98	9046
Amusement	0.96	0.99	0.98	6746
Meditation	0.98	0.99	0.98	7510
Accuracy	-	-	0.98	60,000
Macro Avg	0.98	0.99	0.98	60,000
Weighted Avg	0.98	0.98	0.98	60,000

**Table 7:** Comparison with previous techniques.

Authors	Techniques	Accuracy	Precision	Recall	F1-Score
[55]	CNN	87.7	NA	Na	NA
[24]	ML techniques	NA	NA	NA	83.34
[23]	ML techniques	80.0	NA	NA	NA
Proposed Approach	FL-DNN	99.1	98.0	97.0	97.0

## Data Availability

Not applicable.
